# Genomic analysis of *Enterococcus durans* NT21, a putative bacteriocin-producing isolate 

**DOI:** 10.22099/mbrc.2022.44088.1760

**Published:** 2022

**Authors:** Nashwa Tarek, Ahmed O. El-Gendy, Ahmed S. Khairalla, Medhat Abdel-Fattah, Eman Tawfik, Ahmed F. Azmy

**Affiliations:** 1 *Department of Botany and Microbiology, Faculty of Science, Beni-Suef University, Egypt*; 2 *Microbiology and Immunology Department, Faculty of Pharmacy, Beni-Suef University *; 3 *Department of Biology, University of Regina, Saskatchewan, Canada *; 4 *Basic Science Department, Faculty of Oral and Dental medicine, Nahda University Beni-Suef (NUB), Beni *; 5 *Department of Botany and Microbiology,* *Faculty of Science, Helwan University, Egypt*; ¥ * Eman Tawfik and Ahmed F. Azmy contributed equally to the project.*

**Keywords:** Whole-genomic sequencing (WGS), *Enterococcus durans *NT21, Probiotics, human oral cavity, Crisper-Cas

## Abstract

*Enterococcus* species are a long-standing and non-pathogenic commensal bacterium, representing an important part of the normal. *Enterococcus durans* is a rarely isolated species from animals and humans, and it was a tiny constituent of human oral cavity and animal intestinal flora, as well as animal-derived foods, particularly dairy products. This study evaluated the security of our strain *E. durans* NT21 by using whole-genome sequencing (WGS), physicochemical features, and antimicrobial activity. The complete genomic of our strain *Enterococcus durans* NT21was sequenced and analyzed by using several bioinformatics tools to identify bacteriocin genes, virulence genes, antibiotic resistance genes, Crispr-Cas and pathogenicity islands. The results showed that our strain NT21 lacks the presence of virulence genes, pathogenicity islands, plasmids and has only two antibiotic resistance genes. On the other hand, it produces three bacteriocin-like inhibitory substances (Enterolysin A, P and L50a). It has six gene-encoded Crisper-Cas and one cluster Crispr-Cas gene. According to our findings, *E. durans* NT21 is a possible probiotic strain that is safe for both human and animal use.

## INTRODUCTION

Lactic acid bacteria (LAB) are a common bacterial species representing a large scale of normal human flora, and they can ferment carbohydrates for energy [1]. *Enterococci spp.* are a group of lactic acid bacteria (LAB) [2-4]. Several strains of *Enterococcus* have been found to produce antimicrobial compounds such as bacteriocins [5]. These antimicrobial peptides (bacteriocins) are of particular interest because they have the potential to be used in the pharmaceutical sector and serve as safeners against the growth of undesirable microbes [6, 7]. Diverse and numerous strains of *enterococcus* are represented a main constituents of the human body because they play a role in biochemical and biological needs for survival, development, reproduction, and physiological processes, and also the recycling of nitrogenous components and the metabolism of amino acids (AAs) are supported by enterococcus microbes in the gut [8].

 Probiotic bacteria have been seen to maintain normal intestinal and oral microflora [9], lowering cholesterol levels, and also change immune responses in both human and animals. It is also defined as "a living microbe that, when given in modest amounts, has health benefits to the host." [10-12]. It is clear that many probiotic bacteria are involved in the synthesis of some vitamins [13]. In this study, recent advances in probiotic potential, safety ultilization and knowledge of bacterion production form our strain will be studied, focusing on their poteintail application changes in different fields by using the complete genomic analysis and functional annotation of this stain *E. durans* NT21. 

## MATERIALS AND METHODS

**Bacterial strains collection, isolation and identification growth conditions:** About 100 oral swabs from healthy individuals were collected from March 2019 to May 2019 from Oral and Dental hospital-Nahda University. All swabs were aseptically transported by Amie’s transport medium [14]. Each collected samples were submitted to the microbiology laboratory at Nahda University (Beni-Suef, Egypt) for cultivation and testing. Swabs were inoculated aerobically in MRS broth media (Oxoid, U.K) for 24 hr at 37^O^C. The pure culture of the isolates cultures preserved as a frozen stock at –80℃ in 20% (v/v) glycerol [15].

**Screening for production of bacteriocin and bacteriocin like substances: **The agar diffusion technique evaluated the production of bacteriocin and bacteriocin-like substances of candidate isolates [16]. Briefly, about 70 μl of an overnight culture of *Micrococcus luteus ATCC 10240*, the most sensitive indicator strain, was mixed with 5 ml of molten MRS soft agar (45°C) and then poured into a petri dish containing 10 ml solidified MRS base agar as reported by [17]. Wells (cups) with 0.5 cm diameter were made using a hollow tube. In each well, 50 μl of the supernatant containing the bacteriocin of each isolate was added as mentioned by [18]. The activity of bacteriocins were revealed by the formation of indicator strain's inhibition zones (halos) [18]. Finally, from the previous data, we selected our *Enterococcus sp.* NT21 to study its genomic features, metabolic potential and probiotic potentiality because it was the most active bacteriocin producing strain.

**Spectrum of activity of bacteriocin and bacteriocin like substances from isolated** ***Enterococcus*** **spp:** *Enterococcus sp.* NT21 was inoculated (1% v/v) into MRS broth and then incubated aerobically for 24 h at 37℃. The fermented broth was centrifuged at 5,000 × g for 10, and then was collected [19]. The pH of the CFS was adjusted to 6.5 to prevent the inhibitory effects of organic acids and hydrogen peroxide (H_2_O_2_).as mentioned [18]. The antimicrobial assay was performed against different microorganisms by using the agar diffusion assay as described by [20]. Subsequently, a 50 µl aliquot of CFU was added to wells (6 mm in diameter) pre-inoculated with 10^5^ CFU/ml indicator microorganisms. After pre-diffusion at 4°C for 2 hours, the plates were incubated at 37°C for 24 hours to grow the target microorganisms [17]. The indicator microorganisms used in this study and their growth conditions are listed in Table 1.

**Characterization and physicochemical properties of the bacteriocin-like inhibitory substance (BLIS) in CFS: **Sensitivity of bacteriocin NT21 sample to some hydrolytic enzymes was detected by treatment with RNAase enzyme, α-chymotrypsin, α-amylase and proteinase K. These enzymes were diluted, sterilized, and finally added to CFS samples as mentioned by [18]. Heat resistance of the bacteriocin NT21 were detected by heating the bacteriocin NT21 to 55℃, 80℃, 100℃ and 121℃ [18]. The percentage of activity was calculated by measuring the inhibition zone of the treated sample. The CFS samples were tested for their ability to withstand acidic and alkaline conditions as mentioned [21]. To determine the effect of dithiothreitol (DTT) on the BLIS activity, the CFS sample was incubated as described by [22]. 

**Molecular identification of the NT21 isolate:** This technique includes DNA isolation from the bacterial strain then apply PCR using 16SrRNA primers for identification followed by whole genome sequencing for the product and finally assembly the resulted sequence on NCBI. Genomic DNA was extracted using a bacterial genomic DNA extraction kit (Thermo scientific, USA) as the manufacturer’s protocol. DNA quantification was performed using the Nanodrop ND1000 (Thermo Scientific, USA). The 16S rRNA gene was amplified [23] and purified using eubacteria -specific primers 27F and 1492R as described by [24]. Both strands were sequenced as described in a previous study [25]. High quality DNA was sequenced by Illumina TruSeq Nano DNA platform using 2x151 paired end libraries [26]. The reads were extracted in FASTA format bearing DNA sequence with respective quality values for each base. The genome of *Enterococcus durans *ATCC 6056 (GenBank accession number: ASWM01000000) was used as a reference. 

**Bioinformatics analysis and Functional annotation (Genome characterization):** The functional annotation of the strain NT21became executed by using both PATRIC databases (Patho systems Resource Integration Center) [27] and RAST-SEED server (Rapid Annotation Subsystem Technology) [28]. Additional genomic databases have been looked for genes related to antibiotic resistance databases and virulence genes were downloaded from the Center for Genomic Epidemiology (CGE) [29], CRISPR-cas region prediction (CRISRP finder) [30], pathogenicity islands (PAIDB v2.0) [31]. The map was constructed by using The CGView analysis [32]. The bacteriocin mining tool web-based version 4 (BAGEL 4) was used as an especially valuable resource, to detect the bacteriocin gene cluster [33]. This whole genome shotgun project has been deposited at GenBank under the accession number JAFBBA000000000.

**Statistical Analysis** **: **The data were analyzed using Minitab 19 to find out the means and standard deviation for the different replicates of each treatment. 

## RESULTS

The growth on MRS & bile esculin agar showed that 70 strains from the 100 isolates were belonged to *Enterococcus* spp. These 70 isolates were screened for bacteriocin production using two different techniques: Spot on lawn assay and Cup Diffusion assay. The data showed that only 17 isolates produce bacteriocin or bacteriocin like substances. Based on the phenotypic properties, spectrum pf antimicrobial activity and zone of inhibition; one strain was selected for complete genomic analysis which was the most potent isolate. 

In this study, the data of our strain confirmed antimicrobial activity against 10 indicator bacterial strains. This strain NT021 showed highly antibacterial activity toward Gram-positive indicator bacteria, including methicillin-resistant *S. aureus* (MRSA), *Listeria innocua*, *Streptococcus mutans* and *Micrococcus luteus*. Additionally, it has an inhibitory effect on Gram-negative bacteria like *E.coli* and *Salmonella typhi* ATCC 55669 as mentioned in Table 1.

**Figure 1:**
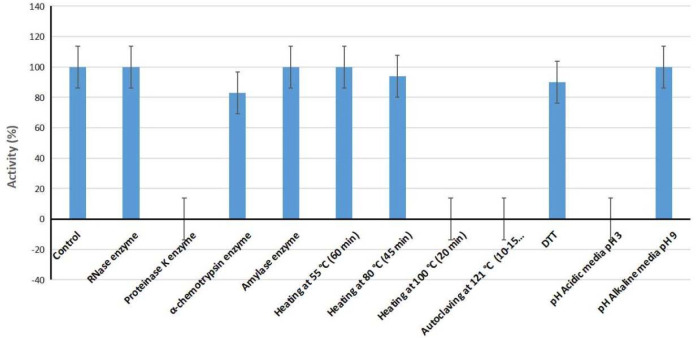
Results for the experiments conducted for characterization of physicochemical properties of *E. durans* NT21. This study showed the stability of bacteriocin NT21 against different conditions of hydrolytic enzymes (RNAase enzyme, proteinase enzyme, α-chemotrypsin enzyme and amylase enzyme), PH (acidic media 3 and alkaline media 9), temperature (heating to 55℃, 80℃, 100℃, and 121℃) and DTT. *Staphylococcus aureus *ATCC 25923 **(MRSA) **used as control. The percentage of activity was calculated (% activity=inhibition zone of the treated sample/inhibition zone of the control sample×100

The proteinaceous nature of the antibacterial compound was determined in our work by treating crude BLIS with proteolytic enzymes. According to our outcomes, BLIS activity was completely abrogated after treatment with the proteinase K enzyme, while the activity was decreased by about 17 % after treatment with the α-chemotrypsin enzyme. Conversely, when CFS was treated with both RNase and Amylase enzymes, BLIS activity was completely stabilized compared to control. The crude BLIS activity was stable at 55℃ for 30 minutes, while the activity decreased by 6% at 80℃. In contrast, it was found that the activity was completely lost at 100℃ and 121℃. According to our data, the activity was totally lost at acidic pH, while it was found to be completely stable at alkaline pH compared to BLIS control. After the treatment with DTT, the activity of our strain was decreased by 10% as metioned in Figure 1 and (Supplementary Table (S1).

**Table 1:** Indicator Strain, and their Growth conditions

**Species**		**Strain**	**Growth conditions**		**Activity**
			**Medium**	**Temperature (°C)**	
*Staphylococcus aureus * **(MRSA)**	ATCC 25923	BHI	37	+++++
*Streptococcus mutans*	ATCC 25175	DSM medium 92	37	++
*Streptococcus pyogenes*	ATCC 19615	DSM medium 693	37	+
*Escherichia coli *	ATCC 5087	BHI	37	+
*Micrococcus luteus*	ATCC 10240	BHI	37	++
*Staphylococcus aureus * **(MRSA)**	LMGT 3242	BHI	37	+
*Lactobacillus plantarum *	LMGT 2003	BHI	37	++
*Listeria innocua *	LMGT 2710	BHI	37	+
*Enterococcus faecalis *	V583	BHI	37	+++
*Salmonella typhi *	ATCC 55669	BHI	37	+

(+), (++), (+++), and (++++) mens that inhibition zone equal to 5-10, 10-15, 15-20; and more than 20 mm, respectively.

**Figure 2:**
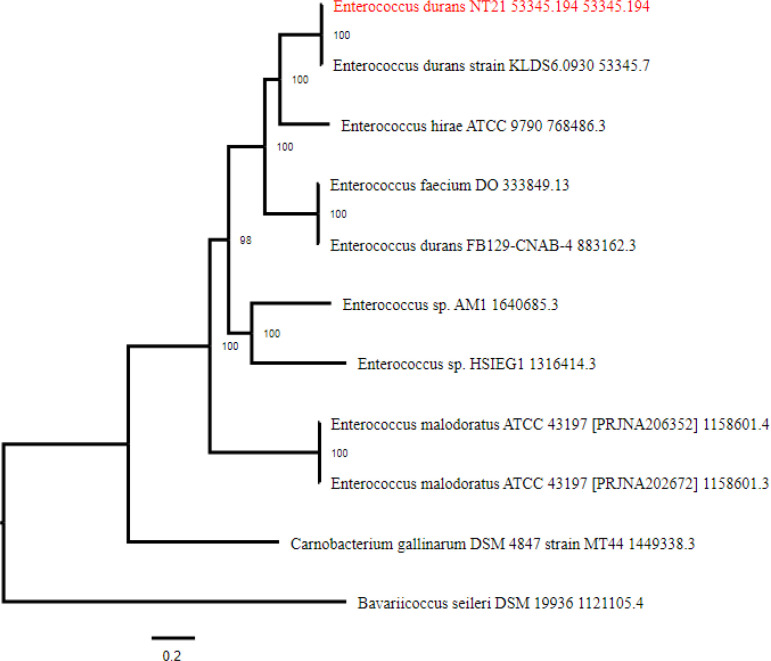
Phylogenetic tree constructed using single-nucleotide polymorphisms (SNPs) in the studied strain's center gene, with intently linked taxa mainly based on 16S rRNA gene sequences. The neighbor joining strategy had been used to create the phylogenetic tree (by using PATRIC database)

**Figure 3:**
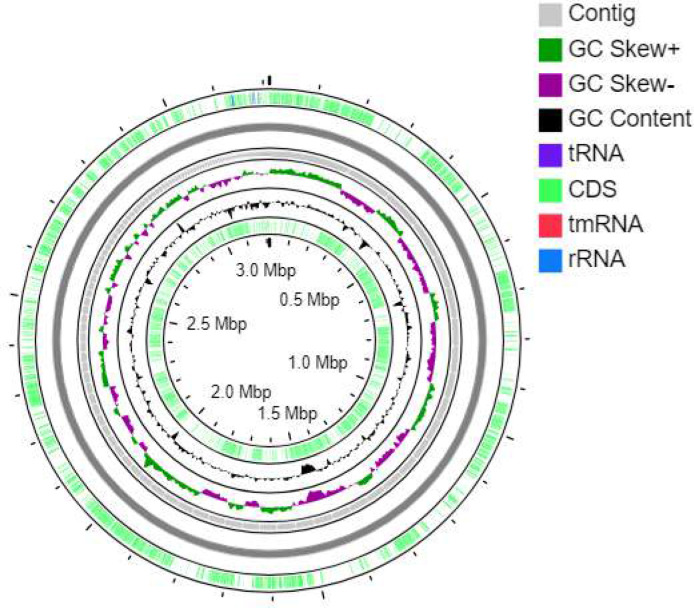
Genome atlas of strain NT04. The atlas depicts a circular view of *Enterococcus durans* NT21's full DNA genome sequence. This circle was created using the server CG viewer. The innermost circle 1 and circle 6 represent CDS (green). Circle 2, show GC content. Circle 3, represents GC skew+ (green) and GC skew-(violet). Circle 4, show contigs (gray)

According to the whole genomic sequencing analysis and 16S rRNA gene sequencing for bacterial identification, our tested strain NT21 is identified as *Enterococcus durans*. So, this strain was named as *E. durans* NT21. The complete genome annotation of *E. durans* NT21 by using RAST server confirmed 1932 genes belonging to 343 subsystems as cofactors, vitamins, DNA metabolism, Fatty acids, protein metabolism, membrane transport, and metabolism of aromatic compounds as mentioned in (supplementary Fig. S1). Using RAST and PATRIC databases, the genome of *E. durans* NT21 consists of a 3,212,931-base pair (bp) with average G+C content of 37.93% and 160 of contigs. Based on the genomic properties of strain NT 21, the coding genes (CDS) are 3.326, contigs L50 are 21, contigs N50 are 48.786, transfer RNAs (tRNA) are 57 and ribosomal RNAs are 3. This whole-genome shotgun project has been deposited at GenBank under the accession number JAFBBA000000000 (Table 2). The phylogenetic tree has been constructed in (Fig. 2) and also the genome atlas is developed in (Fig. 3).

**Table 2:** Genome assembly of *Enterococcus durans *NT21 (using RAST and PATRIC)

**Parameter **	**Data**
Genome size (bp)	3,212,931
No. of contigs	160
N50 (bp)	48,786
GC content (%)	37.63
No. of rRNAs	3
No. of coding sequences	3,326
No. of tRNAs	57
BioProject accession no.	PRJNA698361
GenBank accession no.	JAFBBA000000000
BioSample accession no.	SAMN17715107

**Figure 4:**
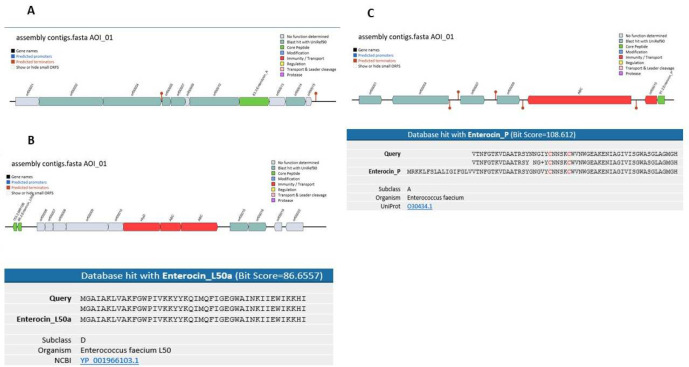
A) Bacteriocin Gene clusters of Enterolysin A; B) Bacteriocin Gene clusters of enterocin_L50a and alignment of the DNA sequences containing the genetic determinants for enterocin _L50a; C) Bacteriocin Gene cluster of enterocin P and alignment of the DNA sequences comprising the enterocin P genetic determinants (using BAGEL 4 software)

The identification of bacteriocin biosynthetic gene clusters was assessed by using BAGEL 4 software. In our strain's genome, the bacteriocin mining software tool BAGEL 4 initially identified three areas of interest (AOIs) containing gene clusters of bacteriocin families: Enterolysin A and Enterocin L50a and Enterocin P (Fig. 4).

According to the Center for Genomic Epidemiology (CGE) database; the genomic analysis showed two antibiotic resistance genes. The gene encoding *ClpL* ATPase proteins (Caseinolytic protease belonging to the AAA+ ATPase superfamily, can be grouped into class I (two ATP-binding domains) was detected in our *E. durans* NT21 strain. The genomic sequencing analysis confirmed that the *clpL *showed a high degree of identity (99.29%) to the *ClpL* gene of *Listeria monocytogenes *strain AT3E plasmid pLM58. The second prevalent antibiotic resistance gene was *aac*(6)-1ih (99.46%) for aminoglycoside antibiotic as mentioned in Table (3). Additionally, we found seven confirmed CRISPR regions, in one of them there were three cas-associated genes (CAS-Type IIC), namely Cas9_1_II, Cas1_0_II and Cas2_0_IIA. The analysis of CRISPR regions confirmed that all crispr-cas regions were Cas cluster-genes as showed in Table (4). On the other side, when comparing genomic sequences of the strain with both PAIDB and Center for Genomic Epidemiology, no pathogenicity islands and virulence genes were found, consistent with the RAST database results.

**Table 3:** Properties of the dominant Antibiotic resistance genes

**Gene name **	**Phenotype **	**Accession **	**Position in contig **	**Coverage **	**Identity**
ClpL	Disinfectant resistance	CP023753	1978-4092	100%	99.29%
aac(6')-Iih	antibiotic inactivation (Aminoglycoside Antibiotic)	AJ584701	17444-17995	100%	99.46%

**Table 4:** Occurrence of CRISPR-cas in *E. durans* NT21

**Contige number**	**Element**	**Cas type**	**Gene number**	**Cas genes**
Contige _7	Cas cluster	CAS	1	Cas3_0_I
Contige _20		CAS-TypeIIC	3	Cas9_1_II, Cas1_0_II, Cas2_0_IIA
		CAS	1	Cas2_0_I-II-III
		CAS-TypeIIA	1	Csn2_0_IIA
Contige _21		CAS	1	Cas3_1_I
Contige _26		CAS	1	Cas3_0_I
Contige _66		CAS	1	Cas4_0_I-II

## DISCUSSION

This study investigates data on the probiotic potential of *E. durans* NT21 strain isolated from healthy oral normal flora. We first conducted a complete genomic study to characterize the genomic features and probiotic potential (bacteriocin production) of our strain. We analyzed whether mobile elements such as antibiotic resistance genes, virulence genes, Pathogenicity islands and Crisper-Cas exist in our genome. 

Complete knowledge of genomic sequences can enable accurate genetic analysis of probiotic bacteria. This includes genetic traits that may be associated with beneficial effects and genetic traits that may be associated with unwanted traits. Enterococcus contains strains associated with serious infections, while other strains are part of the mouth, skin, and intestinal symbiotic human microbiota. Some strains, including *E. durans*, have probiotic properties [34].

Previous research has confirmed that the crude's stability of BLIS was achieved below 100°C temperature, whereas after autoclaving at 121°C for 15 min, the BLIS activity was completely lost [35]. In the treatment with pH variations, the bacteriocin lost its activity at acidic pH 3 but remained stable at alkaline pH 9. Furthermore, previous studies showed that the highest growth was also at alkaline pH (7-9) [36]. However, some showed that the best LAB growth was at acidic pH (2-4) [37]. A similar effect of proteinase K after treating crude bacteriocin was confirmed by Ramakrishnan et al, who assessed that bacteriocin showed completely lost its action after being exposed to the proteinase K enzyme [38]. Crude BILS was unaffected by the treatment with RNAase and amylase enzyme as confirmed by Wang and his colleagues [39]. The activity of crude BILS was significantly decreased by the treatment of DTT as mentioned by [40].

From our genomic studies, the genome of our strain* E. durans *expresses multiple bacteriocin genes: Enterolysin A, Enterocin L50a and Enterocin P. Enterolysin A is a group of bacteriocin class III. It was found that the gene encoded of enterolysin A is broadly dispensed through the enterococcal strains. *Enterococcus faecalis* LMG2333 was the first strain reported for the production of Enterolysin A gene [41]. The results confirmed that enterolysin A, isolated from our *E. durans *NT21 strain, was similar to previously reported in *Enterococcus durans *41D (match= 99.75%) [5]. The second bacteriocin in this strain is enterocins L50a (Ent L50a), an unmodified non-pediocin-like enterocins L50 (Ent L50), which is mainly a plasmid-encoded bacteriocin, produced by *E. faecium* F58 [42]. The data was confirmed to this strain that the enterocin L50a (entL50) cluster gene found was to be identical (100% identity) to *Enterococcus faecium* L50 that was reported by [43]. The third type of bacteriocins is enterocin P; it is grouped as subclass of IIa bacteriocin that is created by *Enterococcus faecium* P13, G16 and AA13 which have a vital role in antimicrobial activity against several pathogenic species such as *S. aureus, L. monocytogenes, *and* C. perfringenes *[44]. The resulting data showed that the structural gene encoding Ent p showed high similarity (match=98.15%) to the gene encoding Ent p in *Enterococcus faecium* reported previously [19]. 

In addition, the functional annotation and genomic analysis of our strain *E. durans* NT21 allowed identifying the genes encoding for antibiotic resistance and Crisper-cas. The antibiotic resistance genes were (aac(6′)-Ii) and ClpL. The aac(6′)-Ii gene, which was first discovered only in *Enterococcus faecium* species and was previously proposed as a marker for the identification of *E. faecium* [45]. This gene was encoded for the chromosomally aminoglycoside acetyltransferase AAC (6′) enzyme that is required for the expression of resistance to specific aminoglycosides [46]. 

To protect the genome from invasion by parasite DNA and maintain genome fidelity in a stable ecosystem, there is a mechanism known as a clustered and regularly spaced short palindrome repeat system (CRISPR) [47]. The CRISPR-CAS system, an immune system acquired by prokaryotes against foreign genetic factors and viral infection and also limits the spread of antibiotic resistance genes [48]. CRSPR-Cas-containing cells can invade and digest any foreign DNA as plasmids, transposons, and viruses [49]. The CRISPR-CAS system is classified according to the existence and arrangement of the gene which encodes the affective proteins [50]. The first research which detected the existence of the CRISPR (CAS) system in *E.durans* showed by [51]. In  *Enterococci*, since the presence of CRSPR is inversely related to the gaining of properties like antibiotic resistance, the wide distribution of CRSPR1Cas1 in this species may be related to the lack of virulence [52]. Additionally, enterococci, containing the Type II-A CRISPR system, have been confirmed to be non-pathogenic [53].

## Conflict of Interest:

The authors have no conflict of interest.

## Supplementary materials

**Figure:** Supplementary Table (S1)

**Figure:** Supplementary Fig. S1
