# Supplementary material for: Genomic analysis of Enterococcus durans NT21, a putative bacteriocin-producing isolate
Source: Mol Biol Res Commun. 2022;11(3):143–53. doi: 10.22099/mbrc.2022.44088.1760 (PMC9661671; doi:10.22099/mbrc.2022.44088.1760)
Supplement: Supplementary file 1 [file mbrc-11-143.s1.pdf]

Table S1: Means of the physicochemical properties of *E. durans NT21*

**Physicochemical properties**	**BLIS activity by Inhibition zone diameters (mm)**
Control	18.00 ± 0.00
**Enzymes**	
RNase enzyme	18.07 ± 0.15
Proteinase K enzyme	0.00 ± 0.00
α-chemotrypsin enzyme	15.10 ± 0.20
Amylase enzyme	18.17 ± 0.32
**Temperature, °C (min)**	
Heating at 55 °C (60 min)	18.13 ± 0.21
Heating at 80 °C (45 min)	17.1 ± 0.2
Heating at 100 °C (20 min)	0.00 ± 0.00
Autoclaving at 121 °C (10-15 min)	0.00 ± 0.00
DTT	16.10 ± 0.20
**pH reactivity**	
Acidic media pH 3	0.00 ± 0.00
Alkaline media pH 9	17.97 ± 0.21

The zone of inhibition was expressed as mean ± standard deviation.

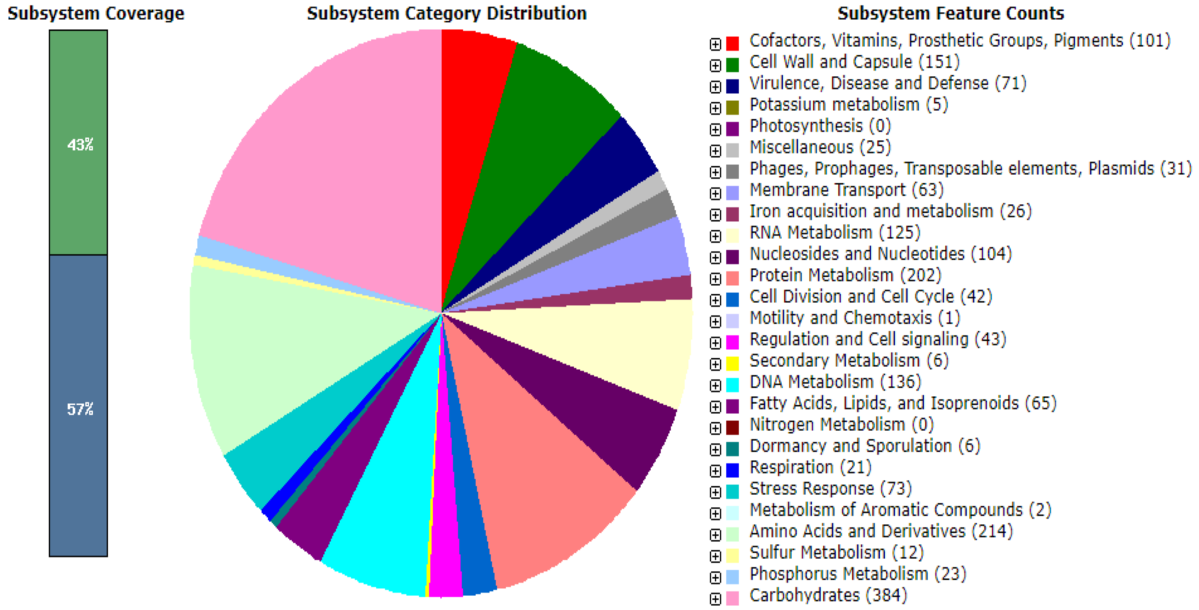

Figure S2: Overview of annotation of *Enterococcus durans* NT21 using RAST database
