# Supplementary figures and images for: Genomic analysis of Enterococcus durans NT21, a putative bacteriocin-producing isolate
Source: Mol Biol Res Commun. 2022;11(3):143–53. doi: 10.22099/mbrc.2022.44088.1760 (PMC9661671; doi:10.22099/mbrc.2022.44088.1760)

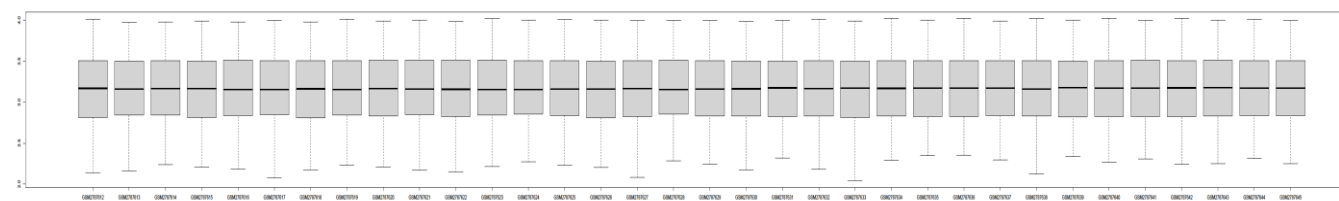

**Figure S1:** Constructed boxplot via Gplot package after normalization

Supplement: Supplementary file 2 [file mbrc-11-143.s2.pdf]
